# Fatal rhinocerebral mucormycosis in a patient with ulcerative colitis receiving azathioprine and corticosteroid 

**DOI:** 10.18502/cmm.5.1.536

**Published:** 2019-03

**Authors:** Narges Najafi, Firoozeh Kermani, Nahid Gholinejad Ghadi, Seyed Reza Aghili, Zahra Seifi, Emmanuel Roilides, Tahereh Shokohi

**Affiliations:** 1 Department of Infectious Diseases, and Antimicrobial Resistance Research Center, Mazandaran University of Medical Sciences, Sari, Iran; 2 Student Research Committee, School of Medicine, Mazandaran University of Medical Sciences, Sari, Iran; 3 Department of Medical Mycology, School of Medicine, Mazandaran University of Medical Sciences, Sari, Iran; 4 Invasive Fungi Research Centre, Mazandaran University of Medical Sciences, Sari, Iran; 5 3rd Department of Pediatrics, Infectious Diseases Section, Faculty of Medicine, Aristotle University School of Health Sciences, Thessaloniki, Greece

**Keywords:** Azathioprine, Corticosteroid, Inflammatory bowel disease, Mucormycosis, Rhinocerebral, Ulcerative colitis

## Abstract

**Background and Purpose:**

Rhinocerebral mucormycosis is a rare fatal fungal infection which is on a growing trend, particularly among immunocompromised patients. Immunosuppressive drugs, including corticosteroids and antimetabolites, increase the risk of this infection. Herein, we reported the case of fulminant rhinocerebral mucormycosis in a patient with ulcerative colitis receiving azathioprine and corticosteroid.

**Case report:**

A 58-year-old woman was admitted to the hospital in a state of coma with an extensive necrosis in her nose. She was afflicted with intestinal bleeding after 1 month of fasting and was treated with azathioprine and a high dose of prednisolone for ulcerative colitis 2 months prior to hospital admission. The direct microscopic examination of the necrotic tissues of the paranasal sinuses showed several non-septate hyphae consistent with Mucorales. Culture media yielded *Rhizopus* species, which was identified as *Rhizopus oryzae* by internal transcribed spacer polymerase chain reaction sequencing. Despite the implementation of surgical and pharmaceutical (liposomal amphotericin B) treatments, the patient expired after 2 weeks of admission.

**Conclusion:**

The gastroenterologists should be aware of the adverse effect of immunosuppressive drugs they prescribe for the treatment of inflammatory bowel disease.

## Introduction

Rhinocerebral mucormycosis is an acute and rare fatal opportunistic fungal infection with rapid invasion in immunocompromised patients [1-3]. The potential risk factors for invasive mucormycosis include diabetes mellitus, solid organ or bone marrow transplantation, malignant hematological disease, human immunodeficiency virus infection, immunodeficiency, and treatment with immunosuppressive drugs (e.g., corticosteroids and antimetabolites) [4-6]. Iron overload, extensive burns, or major trauma are also the potential risk factors for mucormycosis [4, 5]. 

Rhino-orbital-cerebral mucormycosis begins in the palate or paranasal sinuses, progresses to the orbit, and ends in the brain [7]. The early diagnosis and initiation of active antifungal therapy, combined with aggressive surgical debridement, constitute the backbone of a successful treatment [8, 9]. However, even with an optimal therapy, the outcome is not favorable. Herein, we reported a case of fulminant rhinocerebral mucormycosis with ulcerative colitis receiving azathioprine and corticosteroid.

## Case report

On 15 Aug, 2017, a 57-year-old woman with ulcerative colitis, steroid-induced diabetes mellitus, deep vein thrombosis in the lower part of the left thigh, ecchymotic skin lesions, swelling in the left nose, ptosis (i.e., inability to move eyelids), and facial nerve palsy, was admitted to Shafa Hospital in Sari, north of Iran. She was afflicted with intestinal bleeding after Islamic fasting periods (i.e., Ramadan month). 

Ulcerative colitis had been confirmed by clinical manifestations and colon biopsy and was managed with the administration of azathioprine (50 mg/day) and high-dose prednisolone (60 mg/day) for 2 months, prior to hospital admission. Due to prednisolone-induced hyperglycemia, the patient was on treatment with insulin for a month before admission to the hospital. She was also on anticoagulant drugs for the treatment of deep vein thrombosis.

**Figure 1:**
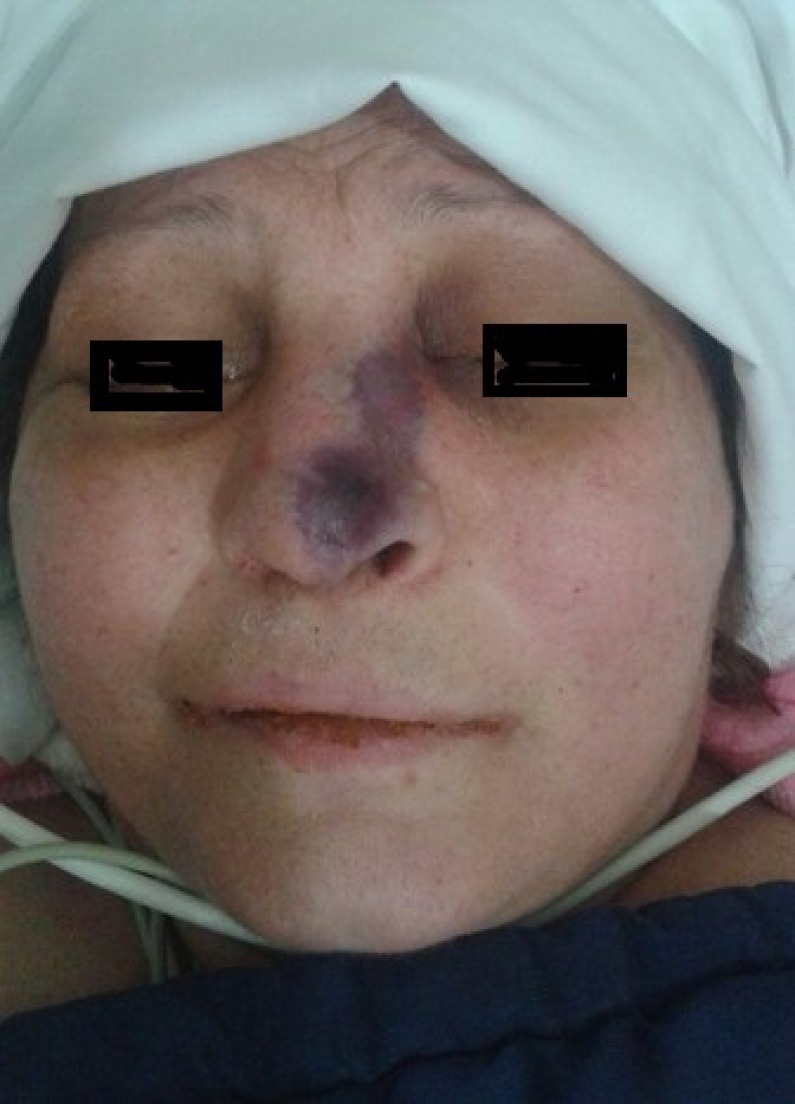
Clinical examination on the second day of hospitalization revealing ecchymotic lesions on the nose skin associated with the edema of the left cheek before the emergence of ophthalmoplegia and ptosis of the left eye

One day after hospitalization, the patient developed progressive periorbital ecchymosis, extensive edema of the nasal area, and nasal ulcer (Figure 1). The eye examination revealed proptosis with 4+ light reaction, evidence of afferent pupillary defect (i.e., Marcus Gunn pupil), no light perception, absolute blindness, ophthalmoplegia, and neurological defects of the cranial nerves 2, 3, 4, and 6.

The results of the laboratory tests and vital signs included a fast blood sugar of 302 mg/dL, white blood cell count of 9460/µl, red blood cell count of 3.53×10^6^/µl, platelets of 60×10^3^/µl, hemoglobin of 8.9 g/dl, blood urea nitrogen of 35 mg/dL, serum creatinine of 2.3 mg/dl, potassium of 2.1 mg/dL, body temperature of 38°C, blood pressure of 120/70 mm/Hg, pulse rate of 80/min, and respiratory rate of 14/min, and glomerular filtration rate of 32. Blood culture and urine culture tests were negative.

On August 18, with regard to the deterioration of the patient's clinical condition and given the high level of suspicion for mucormycosis, the patient was prescribed amphotericin B deoxycholate with a dosage adjustment (0.7 mg/kg/day; 50 mg/day total dosage) coupled with broad-spectrum antibiotics (piperacillin-tazobactam). She was immediately subjected to surgery in which the nasal necrotic tissues were completely removed. The debrided tissues and mucous membranes were immediately sent to the Medical Mycology Laboratory. 

**Figure 2:**
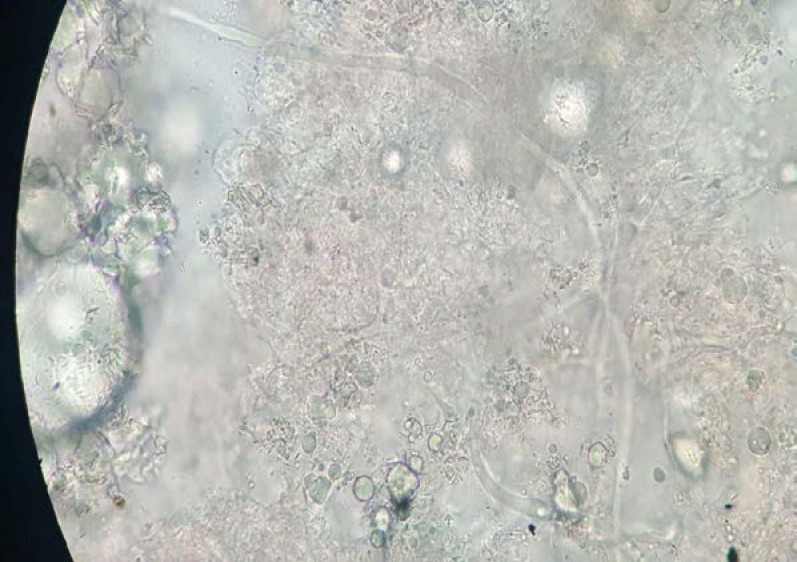
Mycological examination of the biopsy obtained from the maxillary sinus presenting broad aseptate hyphae in the necrotic tissue

The direct examination of the necrotic tissue revealed broad aseptate hyphae with right angle branching compatible to *Mucorales* (Figure 2). The cultures grew gray-brown colonies with cottony texture in which *Rhizopus* species was identified by means of microscopic and macroscopic characteristics. DNA was extracted from the tissue of fresh nasal samples with QIAamp DNA Mini Kit (Qiagen, Hilden, Germany) in accordance with the manufacturer’s instruction. 

The diagnosis was confirmed by polymerase chain reaction (PCR) assay using two universal primers, namely ITS1 and ITS4, to amplify the internal transcribed spacer 1 (ITS1) and ITS2 regions and the 5.8S ribosomal DNA (rDNA) region of the fungi as described previously [9]. The amplicons were sequenced and compared with the GenBank database using the Basic Local Alignment Search Tool, and comparative analysis revealed *Rhizopus oryzae*. The obtained sequences were submitted to GenBank and received the accession number MG946811.

**Figure 3:**
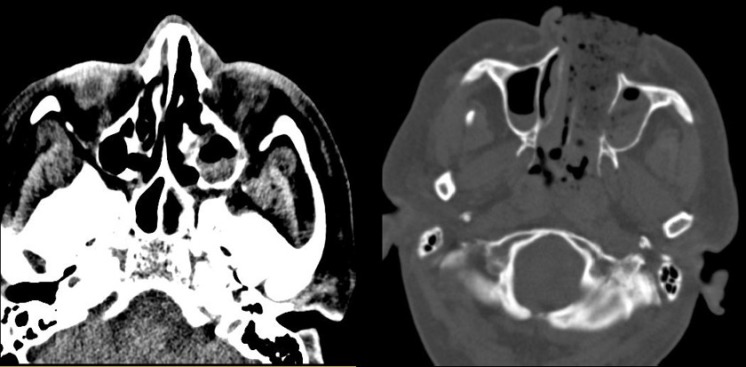
Brain computed tomography scan revealing the involvement of the left maxillary, ethmoidal sinuses, and nasopharynx and the destruction of the bone of the medial wall of the left maxillary and ethmoidal sinuses

The brain magnetic resonance imaging scan revealed the involvement of the maxillary sinus, ethmoid bone, and left sphenoid sinus with no mass effect. The brain computed tomography scan also demonstrated the involvement of the left maxillary, ethmoid sinuses, and nasopharynx, as well as the destruction of the bone of the medial wall of the left maxillary and ethmoid sinuses (Figure 3). 

Given the rapid progression of the disease and the spread of infection to the eyes and brain, the eye removal was recommended. However, the patient’s family refused the enucleation. Consequently, the infection progressed to the eye, and the patient lost her vision. At the same time, severe oral ulcers appeared. Patient’s respiratory and hemodynamic conditions were stable. The dosage of amphotericin B deoxycolate (75 mg/day) increased, and broad-spectrum antibiotics were continued. Eventually, despite antifungal treatment and special care, the patient expired in September 6 due to the rapid progression of the infection to the brain as a result of the refusal of the patient's family regarding enucleation surgery.

***Ethical considerations***

The study protocol was approved by the Ethics Committee of Mazandaran University of Medical Sciences, Sari, Iran (IR.MAZUMS.REC.95.1560). Informed consent was obtained from the patient’s next of kin for each procedure. 

## Discussion

Rhino-orbital-cerebral mucormycosis is a fulminant and potentially fatal disease in immunocompromised patients with uncontrolled diabetes mellitus and corticosteroid therapy [10]. In our case, the use of azathioprine, anticoagulant drugs, and prednisolone for the treatment of ulcerative colitis, followed by the development of hyperglycemia-induced diabetes mellitus, were the predisposing factors of *R. oryzae* invasion in the paranasal sinuses. 

Given the rapid and invasive spread of infection to the adjacent tissues and lack of a rapid and accurate diagnosis, invasive mucormycosis has a high mortality rate (about 85%) in rhinocerebral and disseminated forms (>90%) [11]. Therefore, the correct and early diagnosis of this disease can significantly affect the reduction of these rates.

Hyperglycemia and acidotic condition, iron, and low pH in the blood vessels are favorable for the growth of *R. oryzae *[12, 13]. In our patient, acute metabolic acidosis and fetal bovine serum of up to 300 mg/dL caused by corticosteroid administration had set an ideal condition for the enhancement of the penetration of inhaled *R. oryzae* spores into the nasal tissues. Fever, ecchymotic skin lesions of the nose, facial edema, complete blindness, ophthalmoplegia, proptosis, and neurological defects are the outstanding symptoms of angioinvasion and extension beyond the sinuses [14]. 

The patient's life can be saved from imminent death by surgical debridement and high-dose amphotericin B treatment to prevent the progression of the *R. oryzae* to the brain. Azathioprine is a well-known immunosuppressive agent used for many years [15]. Low doses of azathioprine is effective in the treatment of Crohn's disease and ulcerative colitis, which are the two major types of inflammatory bowel disease (IBD) [16, 17]. Azathioprine inhibits the DNA, RNA, and protein synthesis and suppresses both T- and B- cell production impacting on cellular and humeral immunity. 

Therefore, azathioprine is associated with an increased risk of developing some opportunistic infections, including those caused by cytomegalovirus and varicella-zoster virus, as well as invasive mucormycosis [18-20]. In our case, the long-term usage of azathioprine for the treatment of ulcerative colitis may have predisposed the patient to fulminant rhinocerebral mucormycosis. 

The review of the literature revealed 7 cases of mucormycosis reported in patients with gastrointestinal diseases, such as ulcerative colitis and IBD. Almost all of them were on immunomodulatory therapy with azathioprine, infliximab, and corticosteroids [21]. High levels of glucose increase the expression of glucose-regulated protein 78 (GRP78) at the level of the endothelial cells of the sinus, lungs, and brain that binds to Mucorales germlings and plays a role in the invasion and expansion of the infection [13]. In our case, the previous usage of a high dose of corticosteroids may have increased the expression of GRP78, thereby causing rhinocerebral mucormycosis. 

The cornerstones of the treatment of rhinocerebral mucormycosis are early surgical debridement and suitable antifungal therapy [22]. In the present case, the patient's family did not agree to perform fororbital exenteration surgery despite the clinician’s explanation about the patient's condition and the importance of surgical debridment in the outcome. Therefore, the necrotic tissues that preclude the sufficient delivery and penetration of the antifungals into the affected area may have led to further invasion to the periorbital and neurovasculature areas resulting in death. Antifungal prophylaxis is recommended for the prevention of invasive fungal infection in pre-transplant period; however, it remains uncertain for other high-risk groups [23].

## Conclusion

This report is helpful in encouraging the gastroenterologists to pay attention to the possibility of invasive mucormycosis after prescribing immune-suppressive drugs, importance of the dosage adjustment of anti-IBD agents, and careful observation by making repeated ear, nose, and throat visits. Consequently, it is recommended to consider early diagnosis, appropriate antifungal treatment, and adjunctive surgical debridement in the patients with fulminant rhinocerebral symptoms.
